# 1957. Increased short-term vaccine effectiveness of BNT162b2 with same-arm vs. cross-arm administration of sequential doses

**DOI:** 10.1093/ofid/ofac492.1583

**Published:** 2022-12-15

**Authors:** Daniel Grupel, Yehonatan Pasternak, Yochai Schonmann

**Affiliations:** Soroka University Medical Center and Ben Gurion University of the Negev, Beer Sheva, HaDarom, Israel; Schneider Children's Medical Centre in Israel, Kipper Institution of Allergy and Immunology, Sackler School of Medicine, Tel Aviv University, Petah Tikva, Israel, Petach Tikva, HaMerkaz, Israel; Clalit Health Services, Tel Aviv, Israel and Ben Gurion University of the Negev, Faculty of Health Sciences, Beer Sheva, Israel, Beer Sheva, HaDarom, Israel

## Abstract

**Background:**

It is unknown whether administering the second BNT162B2 vaccine dose on the cross-arm or the same arm as the first dose creates a more robust local and systemic immune response leading to favorable clinical results. We used data from Israel’s largest healthcare provider (Clalit health services [CHS]) to assess the impact on vaccine effectiveness of contralateral vs. ipsilateral administration of BNT162b2.

**Methods:**

A retrospective cohort study, conducted on all CHS members who received the BNT162b2 vaccine between December 2020 and December 2021.

The primary endpoint was a positive RT-PCR test for SARS-CoV-2 between 10 days and 38 days after the administration of the second dose of bnt162b2.

A logistic regression model was used to compare the likelihood of COVID-19 infection at different time intervals following vaccination.

**Results:**

During the study, 2,678,226 CHS members received both doses of BNT162b and were eligible for analysis. Of these, 2,367,694 (88.41%) received the first two doses of the vaccine on the same arm (ipsilateral). The primary endpoint was observed in 2061 (0.077%) participants. The primary endpoint was observed more frequently in the contralateral vs. the ipsilateral group. The adjusted odds ratio for occurrence of the primary endpoint in the ipsilateral vs. the contralateral group was 0.83 (95% CI 0.73-0.94 P=0.004).

Statistically significant reduction in OR was also observed in two of our secondary outcomes – hospitalization due to COVID-19 and all-cause mortality.

**Figure:**
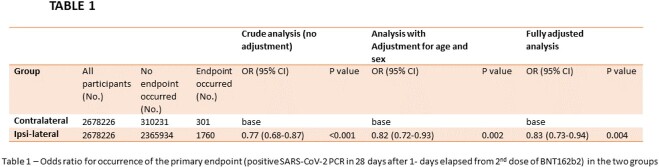

**Conclusion:**

Our study suggests that administration of the first and second BNT162b doses in the same arm, might increase vaccine effectiveness in the short term, possibly due to more robust local lymph node activation. This intervention could have a dramatic effect on public health. Further studies are needed to assess the long-term effectiveness of our findings.

**Disclosures:**

**All Authors**: No reported disclosures.

